# Reese’s Formulary

**Published:** 1850-11

**Authors:** 


### ARTICLE II.

#### REESE'S MEDICAL FORMULARY.

*The American Medical Formulary; based upon the United States and British Pharmacopœias; including also numerous standard formulæ, derived from American and European authorities. Together with the medical properties and uses of Medicines; Poisons, their antidotes, tests; etc. Designed for the Medical Pharmaceutical student.*—By JOHN J. REESE, M.D., Lecturer on Materia Medica in the Philadelphia Medical Institute, Fellow of the College of Physicians. etc. Philadelphia: Lindsay & Blakiston, 1850; pp. 357, 12mo. (From the Publishers, through S. C. Griggs & Co.)

The title of this little book renders it unnecessary for us to enlarge on the character of its contents; and we would no more think of reviewing it, than we would of criticising the multiplication table. It is one of the series of works know as the *Medical Practitioner's and Student's Library.* M.

